# Supplementary material for: A goalkeeper’s performance in stopping free kicks reduces when the defensive wall blocks their initial view of the ball
Source: PLoS One. 2020 Dec 31;15(12):e0243287. doi: 10.1371/journal.pone.0243287 (PMC7774851; doi:10.1371/journal.pone.0243287)
Supplement: S1 File — This file presents the methods and results of two additional experiments with naïve participants, in which the initial distance (Experiment 2) and initial sideward position (Experiment 3) were varied. (PDF) [file pone.0243287.s001.pdf]

S1 File

Supporting Information: Methods and Results for Experiments 2 and 3

A goalkeeper's performance in stopping free kicks reduces when the defensive wall blocks their initial view of the ball

Theofilos Ch. Valkanidis[1]*, Cathy M. Craig[2, 3], Alan Cummins[1], Joost C. Dessing[1]

[1]School of Psychology, Queen's University Belfast, Northern Ireland, UK.

[2]School of Psychology, Ulster University, Belfast, Northern Ireland, UK.

[3]INCISIV Ltd., 18 Ormeau Avenue, Belfast, Northern Ireland

* Corresponding author

E-mail: tvalkanidis01@qub.ac.uk (TV)

Introduction

In the experiment presented in the main text, only central free kick positions from a single distance were included. In the game of football, free kick locations necessarily vary [1]. We aimed to widen the range of initial ball positions for which we tested the effect of the wall and conducted two additional experiments with naïve participants in which we varied the distance from which the free kick was taken (with fixed ball speed and thus co-varying flight times, Experiment 2) and the sideward position the free kick started position (with constant flight times, Experiment 3).

Methods

In the following, we will describe only those aspects of the methods that differ from the main experiment (see the main manuscript for all the other details).

Participants

Thirty different naïve participants volunteered for Experiments 2 and 3 (15 for each). All participants provided written informed consent prior to the experimental process. Both experiments approved by the Queen's University Belfast Faculty of Engineering Research Ethics Committee (EPS-18-12, EPS-18-01). Part of the data collection was completed in the context of two undergraduate thesis projects; for Experiment 2, this also entailed a single expert goalkeeper, the data of which are not presented/discussed in this study, but available here https://osf.io/s2bc9/ (identifier P8).

Experimental set-up

Experiment 2 involved two different initial ball distances (20m and 26m), exactly in the centre of the goal; average forward ball speed was kept constant, resulting in two flight times (1.0s for the 20m initial distance and 1.3s for the 26m initial distance). We used slightly different arrival heights for the

dummy trajectories, removing the free kicks that hit the bar (1.38m, 1.75m, 2.12m). Dummy trajectories varied in their initial distances (and flight times) as just described, which meant that only a single repetition for each condition was needed to create a total of 180 dummy trials. In the third experiment free kicks originated from two sideward positions (5m left and right of goal centre) both at a 23m forward distance from the goal and we used the same arrival heights for the dummy trajectories as in Experiment 2; again, a single repetition sufficed to create the set of 180 dummy trials. All trajectories in this experiment had a flight time of 1.1s. The wall position was set according to common goalkeeping guidelines: the inside shoulder of the outer defender in the wall was placed along the line from the initial ball position to the goalpost on the same side of the goal and the wall was placed perpendicular to this line.

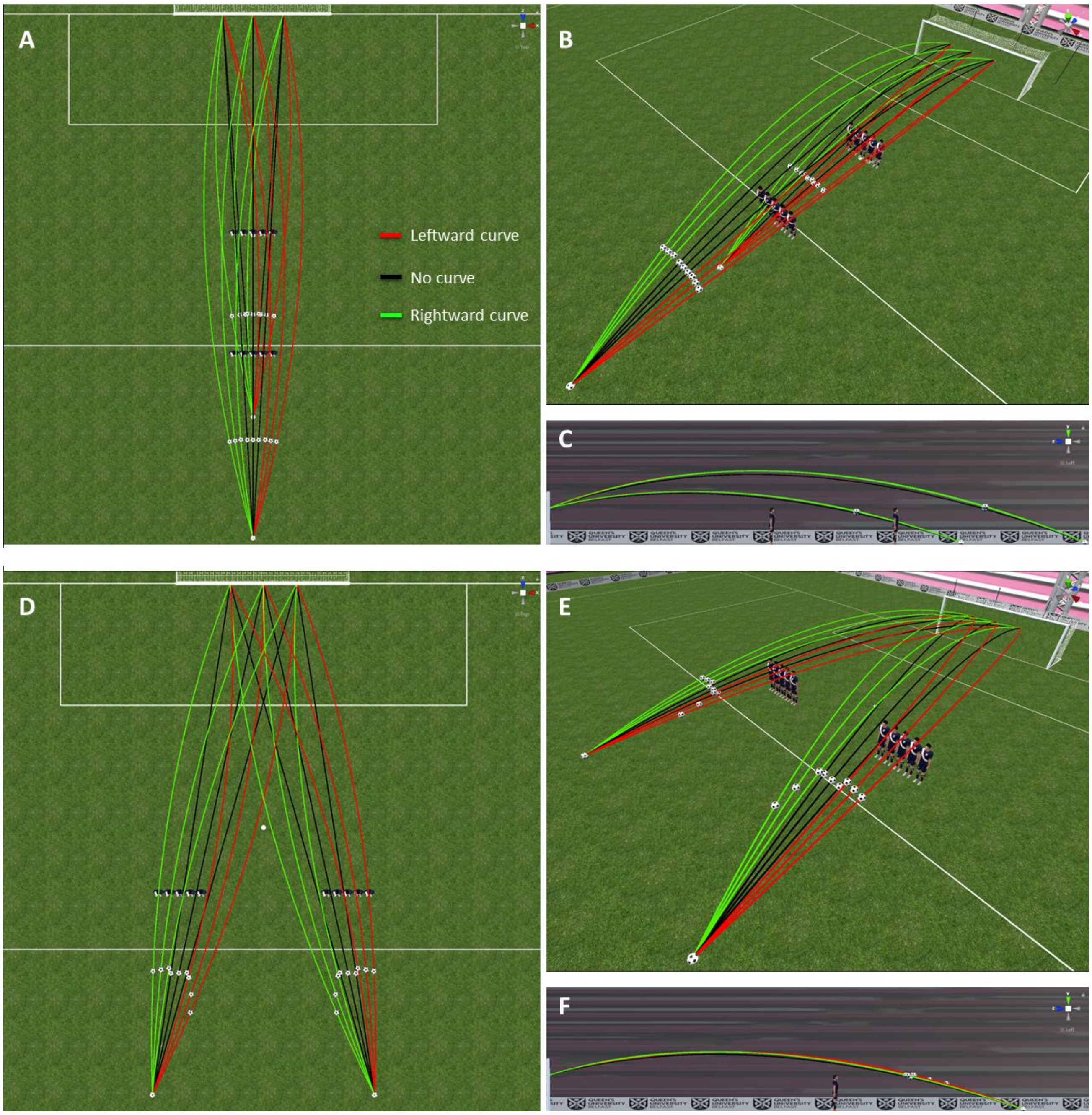

Fig S1. Visual representation of the ball path in the virtual environment in Experiments 2 (A-C) and 3 (D-F). (A, D): top views; (B, E): Diagonal side/top view; (C, F): side view (from the goalkeeper's right). Each panel shows all trajectories (see legend in (A) for colour coding; curve directions expressed from the goalkeeper's perspective), as well as the wall and the ball positions at the frame the entire ball visually cleared the defenders' shoulders. The ball paths are only shown until the goal line. Note that the background (stands) in (C, F) was made darker to more clearly show the paths (see (B, E) for real colour). See Supporting Video S3 and Supporting Video S4 for moving ball trajectories.

Data analyses

All data and analysis code are available in anonymized for at https://osf.io/s2bc9/. Based on the results presented in the main text, we expected a later movement initiation and reduced performance (AE) due to the wall-induced occlusion for both Experiments 2 and 3. The moment of initiation was averaged across all repetitions, final sideward ball positions, curve conditions, and flight times and compared between wall and no wall conditions. For Experiment 2, the effect of the wall on AE was tested separately for the two initial distances/flight times (after averaging across all repetitions, final sideward ball positions and curve conditions). Therefore, for Experiment 2 we adjusted the significance levels for 3 tests (effect of the wall on moment of initiation and its effect on performance for the separate flight times/distances) using a step-down Holm-Šídák procedure (retaining $\alpha = 0.05$ across these tests). For Experiment 3 we had no reason to expect the sideward initial ball distance to modulate the effect of the wall, and thus did not separately test the effect on performance for these conditions. We adjusted the significance levels for two comparisons (effect of the wall on moment of initiation and AE) using a step-down Holm-Šídák procedure (retaining $\alpha = 0.05$ across these tests).

Like in the main experiment, for both experiments we also explored how the behavioural effects of the wall varied across ball trajectories. We compared the effect of the wall on AE between trials requiring movement (arrival positions at ±1.5m) and those that did not (central arrival position), the difference in the effect of the wall on performance between non-curved and curved free kicks, and the wall-induced reduction in the early movement bias in the direction of the curve. Finally, we tested the effect of the wall on the early movement bias separately for congruent and incongruent trials (defined in terms of the direction of curve and the direction of the required movement) and compared the wall-induced performance reduction (for AE) between these congruent and incongruent trials. For these 6 tests per experiment, we adjusted the significance levels using a step-down Holm-Šídák procedure (retaining $\alpha = 0.05$ across these tests within each experiment). For all visualizations of tests involving AE, we also summarized the accompanying success rate; as for the

main experiment, we did not conduct any statistical tests for the success rate. For both experiments, all tests for the moment of initiation and the early movement bias (X_{early}) were two-tailed paired-samples *t*-tests and all tests for AE were two-tailed Wilcoxon signed-rank tests. For a detailed motivation for these procedures we refer the reader back to the main manuscript. Trial exclusion criteria were the same as for the main experiment; on average, 19.6 trials were excluded per participant in Experiment 2, and 35.3 trials per participant in Experiment 3.

Results

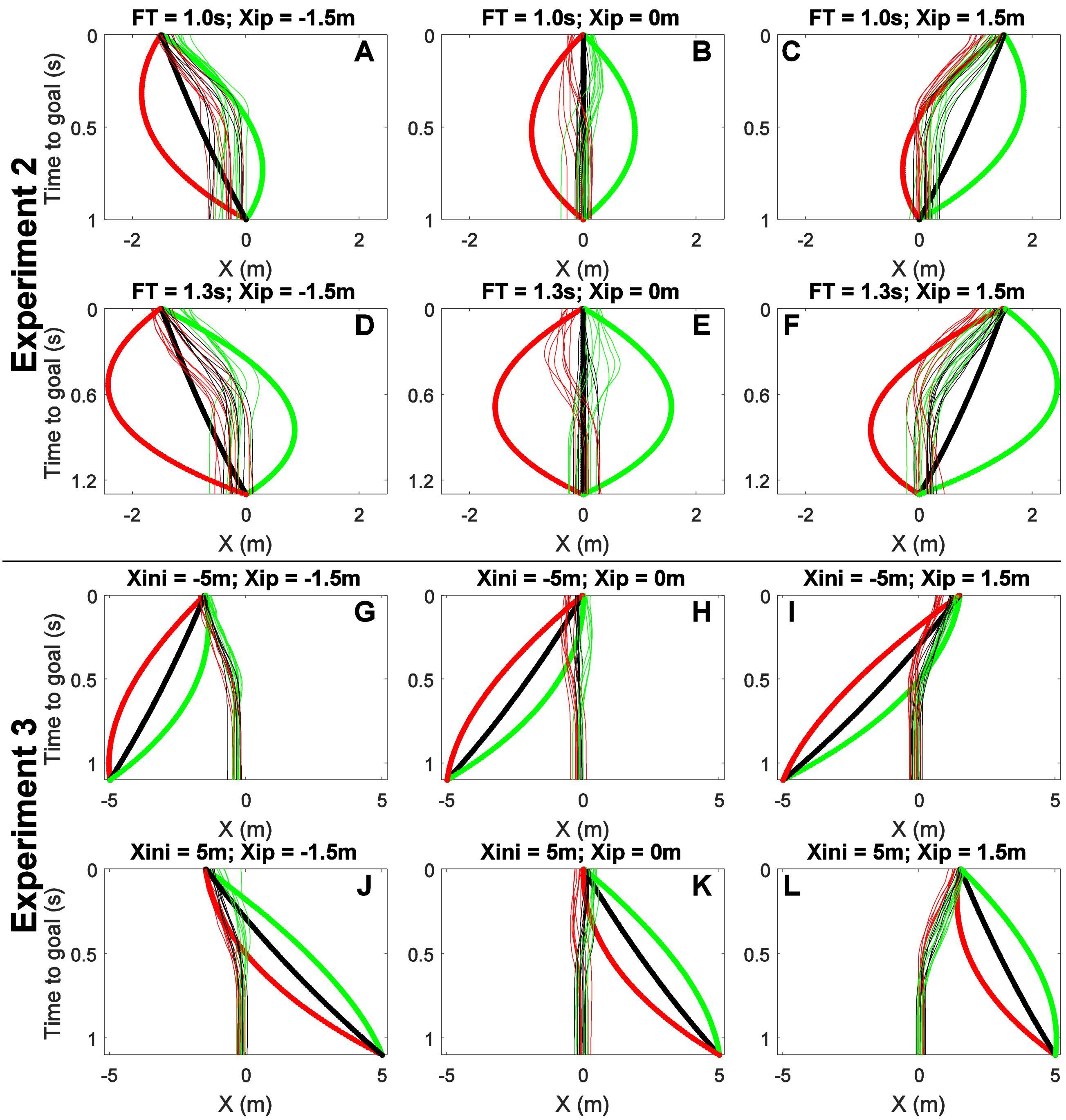

Fig S2 Representative Horizontal hand and ball trajectories. Ball trajectories with different levels of curvature are represented using bold lines of different colours (red= rightward curve; black =(no curve; green = leftward curve) and hand movements are represented using fine lines with the same corresponding colour to match the ball trajectory curvature. The panels depict the lateral ball and hand position as a function of the time before the ball reaches the goal, in the three final positions (Xip= -1.5m[left panels], 0m[middle panels], 1.5m[right panels]). For the Experiment 2 top panels show positions with 1s ball flight time and bottom panels show positions for 1.2s ball flight time. For the Experiment 3 top and bottom panels represent free kicks with 5m left and right initial ball position respectively; these were not necessarily the hands used to determine performance (see main manuscript).

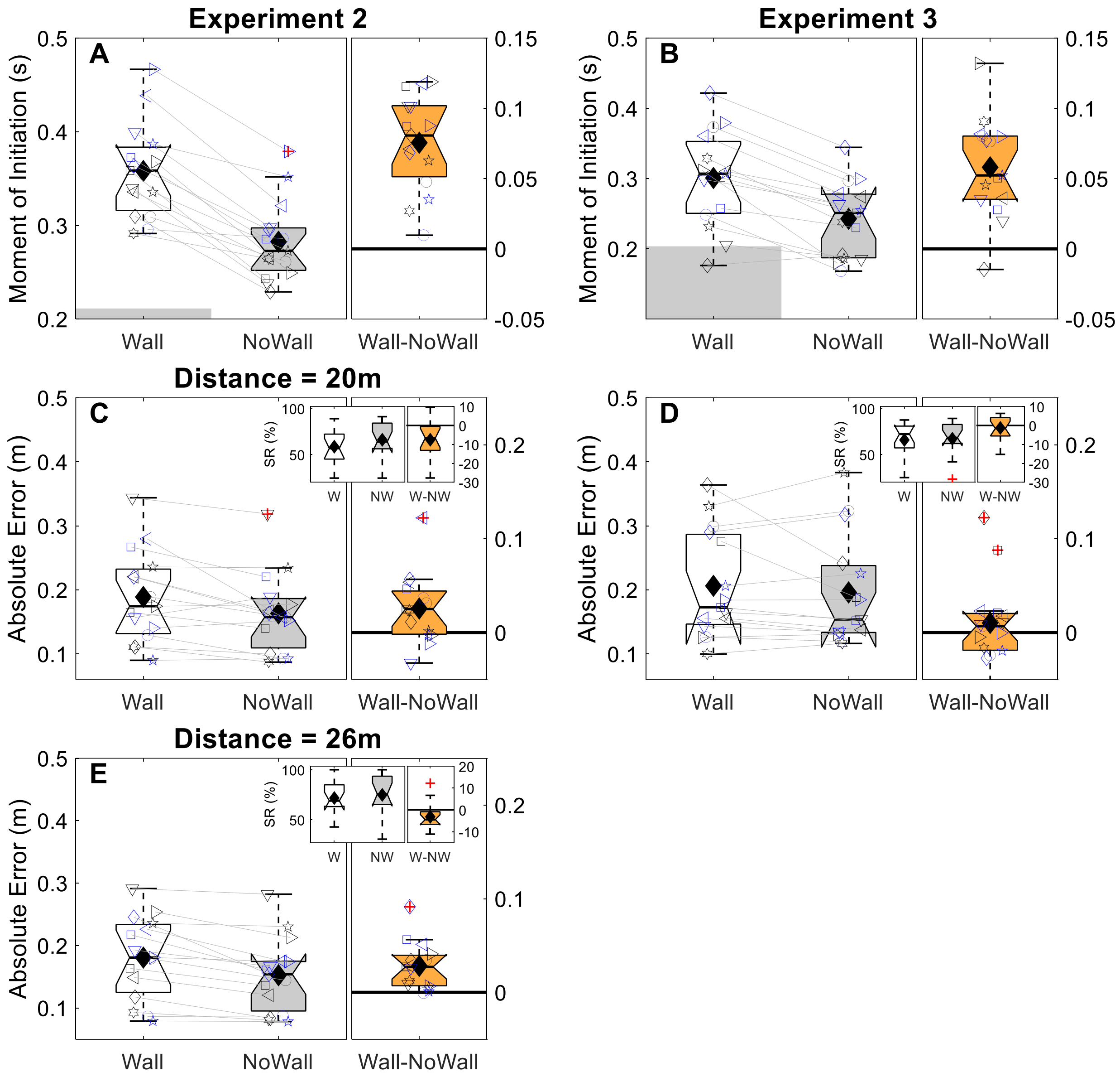

Fig S3. Hypothesis tests for Experiment 2 (left panels) and Experiment 3 (right panels). Panels A and B show the moment of initiation (relative to the moment the ball started moving) averaged across conditions with (white box, "Wall") and without the wall (grey box, "NoWall"), as well as their difference (orange box, axis on the right). The grey shaded area shows when the wall occludes the ball. Panels C and E show the absolute error averaged across conditions with (white box, "Wall") and without the wall (grey box, "NoWall"), as well as their difference (orange box, axis on the right), for the initial ball distances of 20m (C) and 26m (E) in Experiment 2. Panel D shows show the absolute error averaged across conditions with (white box, "Wall") and without the wall (grey box, "NoWall"), as well as their difference (orange box, axis on the right) in Experiment 3. The insets in panels C-E show the success rate (SR in %) in the format of the main panels (W=Wall, NW=NoWall). Formatting of individual data is identical to that described for Fig 4 in the main manuscript.

Experiment 2: Different initial ball distances

Predicted effects of the wall

For this experiment we expected to find an effect of the wall on movement initiation for both initial ball distances, but performance should be mainly affected for the shorter distance. Participants significantly delayed their initiation by 76ms on average ($t(14) = -8.59$, $p_{HS} = 1.8 \cdot 10^{-6}$; Fig S3A). As expected, the occlusion also caused a reduction in performance (AE) for the 20m initial distance ($W = 80$, $p_{HS} = 0.022$; median effect 2.5cm, success rates: No Wall: 66.0%, Wall: 58.5%; Fig S3C). Unexpectedly, we also found that this effect for the far- distance (26m) free kicks, ($W = 118$, $p_{HS} = 2.4 \cdot 10^{-4}$; median effect: 2.7cm, success rates: No Wall: 74.8%, Wall: 71.7%, Fig S3E).

The effect of the wall for different aspects of the free kick

The effect of the wall on AE was significantly larger if sideward movement was required ($W = 94$, $p_{HS} = 0.021$, median difference 2.4cm, success rates: Movement/No Wall: 93.5%, No Movement/Wall: 92.1%, Movement/No Wall: 58.9%, Movement/Wall: 51.6%; Fig S4A). Like in the main experiment, we did not find a significantly different effect of the wall on AE between non-curved and curved trajectories ($W = 38$, $p_{HS} = 0.66$, median difference 1.5cm, success rates: No Curve/No Wall: 80.4%, No Curve/Wall: 75.8%, Curve/No Wall: 65.6%, Curve/Wall: 59.7%). We again found a significant wall-induced reduction of early movement biases in the direction of the curve ($t(14) = 4.08$, $p_{HS} = 0.0056$, mean reduction 2.8cm; Fig S5A). Fig 6A illustrates that a significant difference between wall and no wall was found for congruent trials ($t(14) = 5.92$, $p_{HS} = 2.2 \cdot 10^{-4}$; mean difference 7.0cm), but not for incongruent trials ($t(14) = -0.33$, $p_{HS} = 0.75$, mean difference 0.04cm). Performance did not differ significantly between congruent and incongruent trials (AE: $W = -18$, $p_{HS} = 0.87$, success rates: Congruent/No Wall: 81.3%, Congruent/Wall: 71.1%, Incongruent/No Wall: 24.3%, Incongruent/Wall: 19.9%; Fig S6C).

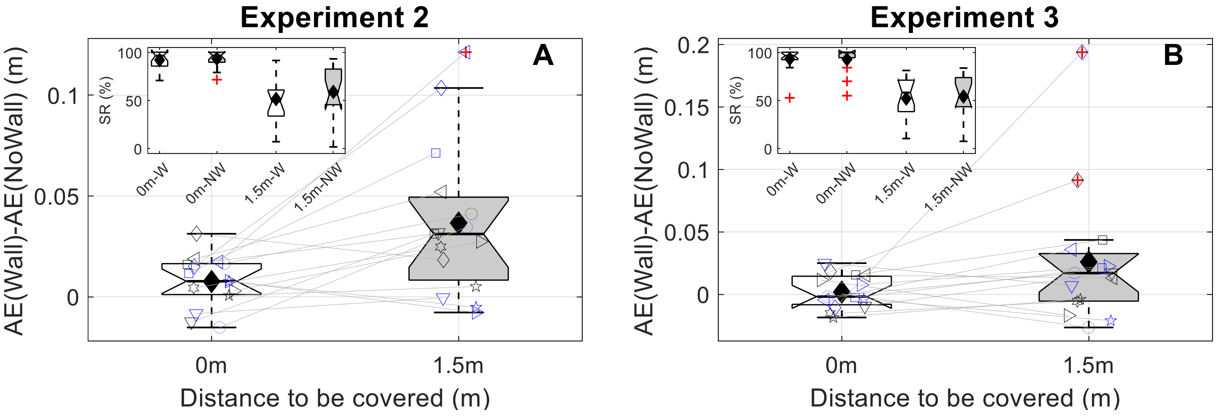

Fig S4. Effect of required movement distance on how the wall affects AE for Experiment 2 (A) and Experiment 3 (B). The difference between the absolute error (AE) averaged for conditions with a wall and that averaged for conditions without a wall is shown for the central final ball position (white box, 0m distance to be covered) and averaged across the outer final ball positions (grey box, 1.5m distance to be covered). Formatting of individual data is identical to that described for Fig 4 in the main manuscript. Insets show the success rates (SR in %) for both distances to be covered for conditions with (0m-W and 1.5m-W) and without a wall (0m-NW and 1.5m-NW); individual data is not shown to avoid crowding.

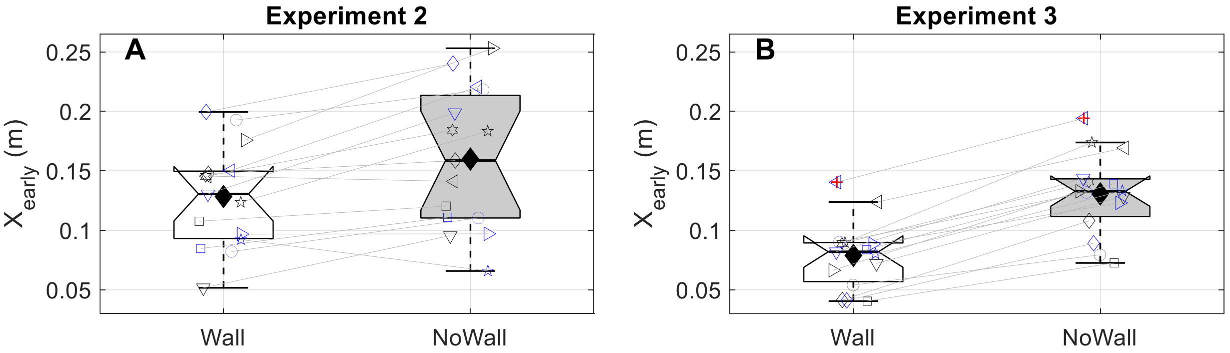

Fig S5. Effect of the wall on the curve-induced early movement bias (X_{early}) for Experiment 2 (A) and Experiment 3 (B). The early movement bias was defined positive in the direction of curve and subsequently averaged across conditions with leftward and rightward curve, separately for conditions with (white box) and without a wall (grey box). Formatting of individual data is identical to that described for Fig 4 in the main manuscript.

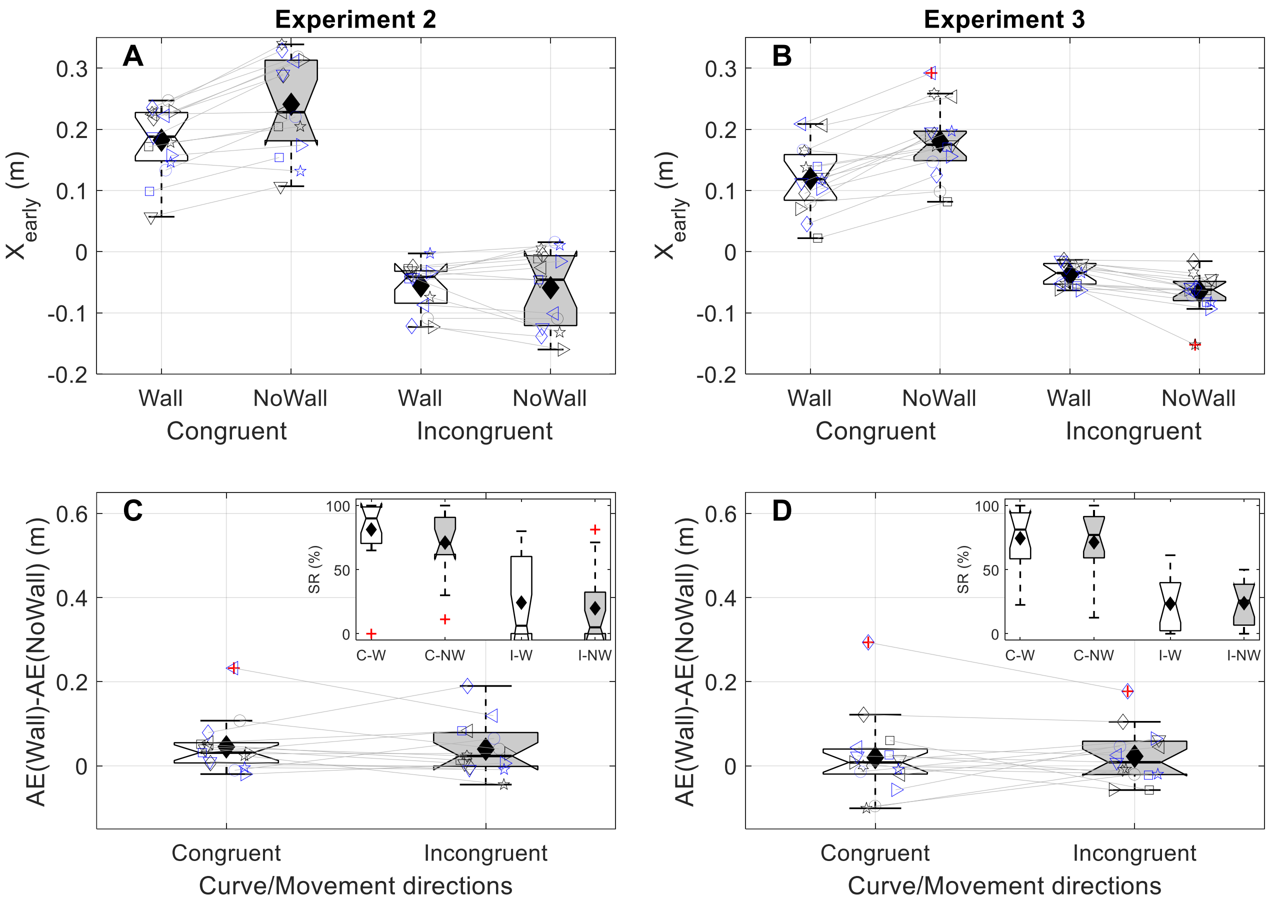

Fig S6. Effect of congruence of directions of curve and required movement on how the wall influenced the interception movement. Top panels (A [Experiment 2], B [Experiment 3]) show the effect of the wall on early movement bias (X_{early}, defined positive in the direction of required movement) and bottom panels (C [Experiment 2], D [Experiment 3]) show the effect of the wall on the absolute error, AE; in all panels the values are averaged across conditions where ball trajectories curved in the direction of the requirement movement (white panels, Congruent) or in opposite direction (grey panels, Incongruent). Formatting of individual data is identical to that described for Fig 4 in the main manuscript. In panels C and D, insets show the success rates (SR in %) for congruent trials with (white box, C-W) and without a wall (grey box, C-NW) and for incongruent trials with (white box, I-W) and without a wall (grey box, I-NW).

Experiment 3: different sideward initial ball positions

Predicted effects of the wall

For this experiment, which included variations in the initial sideward ball position, we tested the hypotheses that the wall induces later movement initiation and affects performance. Participants indeed

significantly delayed their initiation by 58ms on average ($t(14) = -6.32$, $p_{HS} = 3.7 \cdot 10^{-5}$; Fig S2B). However, the effect of the wall-induced occlusion on performance was smaller, less consistent and non-significant ($W = 66$, $p_{HS} = 0.064$; median effect 1.3cm, success rates: No Wall: 67.1%, Wall: 65.8 %; Fig S3D).

The effect of the wall for different aspects of the free kick

The effect of the wall on AE was larger, but not significantly so, if sideward movement was required ($W = 52$, $p_{HS} = 0.39$; median difference: 1.5cm; success rates: Movement/No Wall: 92.9%, No Movement/Wall: 93.4%, Movement/No Wall: 54.2%, Movement/Wall: 52.0%; Fig S4B). Like in the main experiment, the effect of the wall on AE did not differ significantly between non-curved and curved trajectories ($W = -26$, $p_{HS} = 0.74$, median difference -0.5cm, success rates: No Curve/No Wall: 75.5%, No Curve/Wall: 73.1%, Curve/No Wall: 62.9%, Curve/Wall: 62.1%) The wall again reduced the early movement biases in the direction of the curve ($t(14) = 13.37$, $p_{HS} = 1.4 \cdot 10^{-5}$; mean reduction 5.9cm; Fig S5B) and the effect of the wall on the early movement bias differed significantly for congruent trials ($t(14) = 6.6$, $p_{HS} = 5.8 \cdot 10^{-7}$, mean difference -9.4cm Fig S6B). In contrast to the other experiments, however, in Experiment 3 the wall induced a small but significant positive bias for incongruent trials ($t(14) = -4.5$, $p_{HS} = 0.0020$, mean difference 2.5cm Fig S6B). The effect of the wall on performance (AE), however, did not differ significantly between congruent and incongruent trials ($W = 4$, $p_{HS} = 0.93$; median difference 1.2cm, success rates: Congruent/No Wall: 74.5%, Congruent/Wall: 71.4%, Incongruent/No Wall: 23.4%, Incongruent/Wall: 23.7%; Fig S6D).

Discussion

Although many effects were consistent across the experiments, not all of the key findings were. Most strikingly, the effect of the wall on performance (spatial interception error) was smaller and less

consistent (i.e., did not reach significance) in the third experiment, where free kicks started from two sideward initial positions. We think this may relate to several factors. Firstly, while the wall contained the same number of the players, its exact positioning (based on goalkeeping standards) necessarily differed from the first experiments, which affected the occlusion (see Fig 1 in the main manuscript and Video 1). Specifically, the duration of occlusion on average was somewhat shorter than in the main experiment, most clearly so for trajectories that curved in the direction of the required movement (207ms occlusion in the first experiment versus 173ms in the third; incongruent trials: 207ms versus 205ms occlusion). In other words, occlusion was mainly reduced (by ~30ms) only in those conditions for which performance was significant affected by the wall in the main experiment (see Fig 7 in the main manuscript); this would have the effect of reducing the effect of the wall on performance. Secondly, the flight time of the free kicks in the third experiment was 1.1s; our analyses for the main experiment showed that the wall-induced performance reduction was larger for the shorter (1.0s) than the longer (1.2s) flight times. It is quite possible that this effect is not linear (i.e., for large enough flight times no effect would be expected), in which case the effect of the wall for flight times of 1.1s would be smaller than the effect averaged between flight times of 1.0s and 1.2s. However, it should be noted that in the second experiment we did in fact observe a significant wall-induced performance reduction for an even longer flight time (1.3s), which suggests this is not the full story. Clearly, further investigations of this difference are warranted.

References

1. Alcock A. Analysis of direct free kicks in the women's football World Cup 2007. European Journal of Sport Science. 2010; 10:4, 279-284. doi: 10.1080/17461390903515188.
