# Supplementary material for: A goalkeeper’s performance in stopping free kicks reduces when the defensive wall blocks their initial view of the ball
Source: PLoS One. 2020 Dec 31;15(12):e0243287. doi: 10.1371/journal.pone.0243287 (PMC7774851; doi:10.1371/journal.pone.0243287)
Supplement: Attachment — Submitted filename: PlosOneReview.pdf [file pone.0243287.s006.pdf]

Effects of visual occlusion by the wall on goalkeeper performance during free kicks in Football

This paper investigated an interesting topic; visual control of action in football goalkeeping during a free kick with and without a wall. Whilst the paper is generally well written, several sections can be improved as follows. First, a clearer theoretical framework is needed of how early and late visual information maps to guide actions with links to relevant analyses. Second, the manuscript is lengthy, difficult to follow in the disjointed presentation of experiments 2 and 3. In addition, I was not convinced by the use of novice participants in experiments 2 and 3. I would limit the paper to experiment 1. Third, participants need to be accurately categorised as skilled, not experts. Fourth, use of VR technology as reported in the methods is impressive, but I am confused by the omission of kicker kinematics. Fifth, the authors should conduct further work using truly expert goalkeepers before they recommend omission of the wall during free kicks. The wall can influence the kicker's strategy and difficulty of shot execution. Finally, please add a limitations section based upon my comments. More specific details are below.

Specific Comments

Line 29; I was not convinced by the use of novices in experiments 2 and 3. For instance, the authors hypothesise an expertise effect (line 78-79). Goalkeeping is a highly specialised skill, so in order to make practical recommendations the actual expert or skilled performer needs to be studied.

Introduction starts with a professional football example, but does not explicitly state how true expert (or skilled) goalkeepers use visual information to perform to a superior level compared to less-skilled goalkeepers. This is relevant to the hypothesis (line 78-79). Please make this clear in the introduction.

Line 55; sure, but if the wall is omitted the goalkeeper would normally see the kicker's kinematics. This needs to be listed as a limitation. Also, goalkeepers position themselves to a side of the wall to pick-up kicker kinematic cues.

Line 65-72; this section is where a model of early and late visual information that maps onto actions is of value to the manuscript (see Morris-Binelli & Müller, 2017).

Line 78-79; please clearly discuss literature on true expert (or skilled) goalkeepers in order to support this hypothesis.

Line 80-81; I was not convinced as to why the analyses were separated by goalkeeper expertise, when the hypothesis clearly states as expertise effect. A factorial ANOVA is needed.

Line 97; given the available technology and space, I am not sure why the kicker's kinematics was not also simulated. This needs to be listed as a limitation. Both kinematic and ball flight information would make the task more presentative (see Pinder, Davids, Renshaw, & Araújo, 2011).

Line 219; why is skill level not part of these predictions?

Line 244; not sure why success rate could not be analysed using a statistical test?

Line 264-267, 291-296; the model mentioned above would help in the interpretation of this data in discussion section (theoretical implication).

Line 310-311; I do not understand why a main analysis would not include skill level as a factor? If not relevant, only study the skilled group, which would provide a stronger argument for your practical recommendation in due course.

Line 324-326; why present the figure if you state its content cannot be appreciated?

Line 339; effect of the wall relative to skill level performance seems the missing analysis.

Line 377; this section onwards is relevant to the model of visual control of action mentioned earlier.

Line 407 para; reference to Table 1?

Line 488; my understanding is that goalkeepers do not stand directly behind the wall. This needs to be discussed as a limitation.

Line 489; theoretical implications.

Line 499-500; is wireless VR an option?

Line 511; expertise effects are discussed, but the introduction content, hypotheses, and analyses related to expertise need to be more clearly aligned and presented.

Line 537 para; this section does not discuss the mechanism of visual control of action. See earlier comments.

Line 552 para; as mentioned earlier, further work is needed with truly expert goalkeepers before strong recommendations can be made. If the wall is omitted, kicker kinematic cues would also become important. This should be mentioned.

Line 578; the wall can influence the strategy of, and challenge, the kicker.

Morris-Binelli, K., & Müller, S. (2017). Advancements to the Understanding of Expert Visual Anticipation Skill in Striking Sports. *Canadian Journal of Behavioural Science, 49*(4), 262-268. doi:10.1037/cbs0000079

Pinder, R. A., Davids, K., Renshaw, I., & Araújo, D. (2011). Manipulating informational constraints shapes movement reorganization in interceptive actions. *Attention, Perception, and Psychophysics, 73*(4), 1242-1254. doi:10.3758/s13414-011-0102-1
