# Supplementary material for: A goalkeeper’s performance in stopping free kicks reduces when the defensive wall blocks their initial view of the ball
Source: PLoS One. 2020 Dec 31;15(12):e0243287. doi: 10.1371/journal.pone.0243287 (PMC7774851; doi:10.1371/journal.pone.0243287)
Supplement: Attachment — Submitted filename: Comment to Authors.docx [file pone.0243287.s007.docx]

**Comment to Authors**

**General Comments**

This manuscript sets out to assess the effects of visual occlusion by the wall on goalkeeping performance from free kicks which is practically relevant as the free-kicks provide a goal scoring opportunity. The use of an immersive, interactive virtual reality system in the current study allows for a significant degree of experimental control.

While the authors have implemented a high degree of scientific rigour there are a number of concerns that need to be addressed. The most notable concerns relate to the generalisability of this research to real-world context. Despite the high degree of experimental control, this research fails to significantly take into consideration the potential actions of the opposing team members which would significantly impact the inclusion/removal of a defensive wall.

The removal of the free kick taker from the virtual reality system is also a point of contention, as the position of the free kick taker prior to taking the free kick can provide the goalkeeper with contextual information on potential arrival destinations. In addition to this the consideration for the action capabilities of the free kick taker if the wall was indeed removed do not seem to be fully addressed. If, as suggested in the conclusion of this paper, the goalkeeper removed the wall the free kick taker may choose a significantly different free kick style presenting trajectories different to those utilised in this study.

This manuscript also needs to be restructured as content that should be in the introduction, methods, and discussion sections often features in the results section. It may be worth considering restructuring the manuscript to sequentially present the three experiments. For an example of this structure style please see Müller, Abernethy & Farrow - DOI: 10.1080/02643290600576595

**Specific Comments**

***Introduction***

L49 – While I don’t disagree with the quality of the free kick example given, taken by Kieran Trippier, I find the reference to players as *“Professional free kick takers”* unusual. Kieran Trippier, the primary example in this research, is a professional football player but the classification as professional free kick taker needs to be reconsidered as he has only scored 5/6 free kicks over the course of 10 seasons as a professional footballer.

L52-55 – To state that goalkeeper performance does not *critically* depend on seeing the free kick taker is a misleading statement. Yes, as identified by the authors online control which could be referred to as prospective control) can be an effective method for goalkeepers to utilise but that does not mean you can disregard the important role of predictive information (see Panchuk and Vickers 2009 [10.1080/02640410903156449](https://doi.org/10.1080/02640410903156449)).

L60-61 – This studies cited to identify that catching performance is negatively impacted when less time is available are conducted using one-handed catching tasks, which differentiate significantly from saving a free-kick, the performance measure used in this study. This needs to be clearly stated.

L69-71 | L78-79 It would be beneficial to directly address what is hypothesised in the introduction more clearly in the discussion section.

***Methods***

L91 – Please clearly identify that it is age you are referring to here.

L92-93 – This is a low threshold of experience to use the term expert. I would suggest using the taxonomy for skill in sport outline in the Routledge Handbook of Sport Expertise by Baker and Farrow in chapter 13 P147 as a guide – see below.

L142-146 – The introduction of dummy trajectories is an excellent addition to the study design and provides valuable variability.

L169-171 – Is there a reason for this hand position. If so, it would be beneficial for readers if this was explained.

L181-182 – On L143 you state that there are 90 unique dummy ball trajectories, 4 trials of each would equate to 360 trials not 180. Can you clarify the number of trials please?

***Research Question***

Is this a sub-heading under data analysis? Is there a reason research questions is placed in this part of the manuscript and not at as null hypothesis at the end of the introduction section?

L222 – Movement initiation can be a deceiving measurement when we consider the potential for individual differences in control strategies (for example see Ijiri et al 2014 – doi: 10.1080/02640414.2014.946073). Movement-onset (initiation) and movement duration can be very different among participants. This, at the very least, needs to be acknowledged as a limitation in the manuscript.

***Statistics***

L304 – I believe this sentence is missing the word “be” - AE to be non-normally.

L311 – Was there a reason for not directly comparing groups?

***Results***

L317 – The results section contains a lot of repetition and descriptive discussion content. The results should only report the finding of the analysis with little to no interpretation. Amend throughout the results section and simply report the findings.

L318-321 | L374-378 | L407-409 | L412-414 | L429-437 – These are prime examples of what I am referring to. The results section is not a place to restate the aims of the research study, provide comparisons to other research studies or provide a rationale for why particular measurements were conducted.

***Discussion***

L500-502 – Restructure this sentence for clarity.

L518-522 – Main Experiment and first experiment are used interchangeably here. Please choose a term and amend throughout for consistency.

L544 – Is there a need for “for experts they did not” to be in brackets here?

L555 – “Flipside” is two words. It may be worth changing this to term to disadvantage/drawback.

L556-561 – Despite identifying that it is something that needs to be examined in future studies, to suggest that free kick takers or soccer/football players rely on the wall to take a free kick and that removing the wall would make the free kick more difficult seems counter-intuitive. The removal of the wall may allow free kick takers to act upon a number of action capabilities available to them.
