# Supplementary material for: A goalkeeper’s performance in stopping free kicks reduces when the defensive wall blocks their initial view of the ball
Source: PLoS One. 2020 Dec 31;15(12):e0243287. doi: 10.1371/journal.pone.0243287 (PMC7774851; doi:10.1371/journal.pone.0243287)
Supplement: Attachment — Submitted filename: Response to Reviewers.docx [file pone.0243287.s008.docx]

**RESPONSES TO REVIEWER COMMENTS**

Note

In all the responses below, we refer to what we previously called “novice goalkeepers” as “naïve participants” and “expert goalkeepers” as “skilled goalkeepers”, even when referring to formulations in the first version of our manuscript. This is reflective of a change we made in the revised manuscript, for which the motivation is provided below.

Reviewer #1: The study presents a novel and appropriate use of VR simulator to investigate the strategic implications of a conventional sports strategy: Goalkeepers blocking free kicks taken over a defensive wall.

*1.1: The technical specifications of the VR set up and calibration is described in exact and replicable detail. However, I would greatly appreciate links to Video 1 (line 141) and Video 2 (152).*

RESPONSE

Apologies for the omission. The text now includes hyperlinks – videos are hosted on our osf page. We will ultimately also upload the videos to the PLOSone file system.

*1.2: The number and arrangement of trials to accommodate variables of ball placement and kick type/trajectory are very thorough. Although I didn’t follow the comment on reducing from 360 to 180 trials (197). “We only analyzed the experimental trials.”*

RESPONSE

We included what we call ‘experimental’ trials – trials that make up the (factorial) design of all combinations of the manipulated factors – and dummy trials – trials whose main function is to present variability in the number of conditions / required actions (including trials that actually hit the bar, so did not require a save). The latter conditions are mainly included to make the entire set of trajectories (and thus the resulting behaviour) a more realistic representation of what may be encountered in practice (e.g., in a training session) and also prevent the players from ‘remembering’ features of the experimental conditions that may bias the results. In the revised manuscript, we have clarified this motivation and the parts alluding to the (removal of) dummy trials (lines 149-155).

*1.3: Reviewer instructions are to focus on research design and statistical analysis, and I do have a few points of clarification or revision. One is that the designation of “Novice Goalkeepers” is not defined very thoroughly (line 91). Are they regular amateur players (mean age 27 years)?*

RESPONSE

We agree with the reviewer that the designation could have been improved/clarified; this is also reflected in the comments of the other reviewers. To clarify, our “novice goalkeepers” really were not goalkeepers as such; they were participants without explicit goalkeeping experience in organised football. They could be football players. Other reviews raised concerns about our designation of experts. Based on this, we now think the best names for our groups are ‘naïve participants’ and ‘skilled goalkeepers’. To address this point, we have clarified the designation of our groups in the manuscript (lines 93-99) and changed all group names as mentioned.

*1.4: Related to that (311), the authors state that, “No explicit group comparison was conducted.” This calls for further explanation and justification as readers expect such comparisons in expert-novice studies.*

REPONSE

This is a very important point, raised by all the reviewers. We deliberately deviated from the standard expert-novice paradigm by not including explicit novice-expert comparisons. One aim was to examine whether the effects of occlusion reported for laboratory-based one-handed catching generalize to the more practical scenario of goalkeeping for free kicks; this warranted a focus on statistical tests aimed at comparisons of wall and no wall conditions; since the previous catching studies did not involve experts catchers, we included a similar group of naïve participants. The possible practical impact (for real-life football) was another aim of the study; this impact required statistical tests of the effect of the wall for skilled goalkeepers. These aims together motivated the inclusion of both naïve participants and skilled goalkeepers without the need for comparing all aspects of their behaviour explicitly/statistically. Nevertheless, as the reviewers note, we did mention reasons to expect that the effect of the wall on goalkeeping performance might be smaller for skilled goalkeepers. We think our use of ‘It is hypothesized’ in this context has suggested this was a key hypothesis of interest – it was not. The main tests of interest all concerned the effects of the wall. Nevertheless, on reflection we agree with the reviewers that a test is warranted. We have thus included the statistical test comparing the effect of the wall between naïve participants and skilled goalkeepers, which showed that the effect of the wall did not significantly differ between these groups (*p* = 0.56), contrary to the expectation.

We want to note one more thing concerning our focus on tests of the effect of the wall. This approach was motivated from a statistical perspective as well. Had we tested for the effects of all our manipulations and their combinations in a factorial statistical test, most tests would be exploratory/unplanned (i.e., conducted without predictions). Such an approach would have massively expanded the number of statistical effects we would evaluate (which would warrant corrections for multiple comparisons [i.e., not testing explicit hypotheses related to the study aim], Cramer et al., 2016; <https://pubmed.ncbi.nlm.nih.gov/26374437/>). Our approach was to minimize the number of statistical tests to only the most relevant ones, while accounting for the inflation of the chance of making Type 1 errors based on the number of tests conducted. This for instance also motivated a more stringent correction based on the aforementioned additional test. We adjusted our Holm-Sidak procedure for 7 (as opposed to 6) tests – this slightly increased the other p‑values, but did not change the test outcomes (i.e., all conclusions concerning differences remained the same). The additional test is now announced in the Introduction (lines 73-78), the Methods (lines 312-313), presented in the Results (lines 354-356), and discussed in the Discussion (lines 468-470, 475-476). Note that we did not include any further group comparisons, since none of these relate to predictions we had or presented (following the motivation just provided).

*1.5: Although the authors refer to statistical significance in the Success Rate within expert or novice groups (31; 478; 514), I would appreciate Table 1 indicating where differences are statistically significant... if only to “thin the heard” of results. Typically, expert-novice research paradigms give weight to differences that reveal expert advantage.*

RESPONSE

After reading this comment, we now appreciate that our formulation on line 31 could be read to suggest that we statistically tested both performance (Absolute Error) AND Success Rate. This was not the case. As described in the response to the preceding comment, our statistical approach was to focus on the analyses of interest, while avoiding unnecessary analyses that would inflate the chance of making Type 1 errors and reduce statistical power. This is particularly relevant for redundant tests (i.e., the DVs Absolute Error and Success Rate both capture Performance and are thus highly related - although AE is a continuous measure and thus more sensitive [whereas Success Rate may be more practically relevant]). We described this in the previous version and in the revised version on lines 236-242:

“Because this measure captures a magnitude, it is thus more sensitive/powerful than (and thus preferred over) Success Rate (the percentage of balls touched by either of the hands across the included repetitions for each condition). Success Rate, however, is an intuitive and practically relevant measure that clearly defines the task goal. For this reason, we do report descriptive statistics of this measure of performance alongside all statistics for AE. To limit the number of statistical tests, we did not conduct any statistical analyses for Success Rate, but it showed virtually identical patterns to AE.”

We have also scanned the manuscript to remove any formulation that might suggest we statistically tested for any effects on Success Rate to avoid the confusion we think gave rise to this comment. Among others, this involved removing the mention of Success Rate from the Abstract and adding a note to Table 1.

*1.6: The key variables in this study seem to be the placement of the ball and the duration/height of ball flight. The authors note that the free-kick modeling required kicks to be higher in order to be presented as slower. This should perhaps be echoed a few times through the analysis, since different free-kick takers can certainly generate greater pace without increasing trajectory.*

RESPONSE

The formulation referred to by the reviewer relates mostly to constraints from physics. Since we constrained distance and final height in the simulations (and in the experimental design), the only physical way to achieve a longer flight time is to increase the peak height of the ball trajectory. The height an actual free kick enters the goal, however, is not constrained, but a consequence of the kick parameters. As the reviewer notes, in practice kickers can vary many parameters of the kick. Still, if you would compare actual kicks from the same distance, to the same height, with the same spin, by definition kicks with longer flight times will have a higher peak. In the revised manuscript, we now mention this ‘difference’ between the simulated free kicks that aligned with our experimental design and the practical reality of shooting free kicks (Line 155-165).

*1.7: This leads to a question that might be addressed at the end under Future Considerations. While the present study thoroughly investigates the effect of a Wall on goalkeepers, it doesn’t address the effect of a wall on free-kick takers. Some of them, freed of the constraint of kicking over/around the wall, may kick with pace and trajectory that are more challenging for goalkeepers.*

RESPONSE

We agree with the reviewer that the wall may have effects on the kicker and have expanded our treatment of this in the Discussion (lines 571-579). Two effects may be expected. Some kickers may be able to deliver a more powerful/challenging shot in absence of a wall – possibly traveling through the space otherwise occupied by the wall; this could be a beneficial effect (for the kicker). Some kickers appear to use the wall for aiming (as anecdotally is reported by them [[https://www.skysports.com/football/news/11095/11310892/watch-gianfranco-zola-joins-jimmy-bullard-for-a-free-kick-masterclass](https://www.skysports.com/football/news/11095/11310892/watch-gianfranco-zola-joins-)]); such kickers might well be negatively affected by removal/absence of the wall. Indeed, we have plans to study the effect of the wall on different types of kickers (e.g., those preferring curved free kicks and those preferring hard, straighter shots).

*1.8: Although reviewer instructions do not ask about interest or implications, I am compelled to note that this study establishes a framework for using VR simulators to investigate and challenge conventional strategies in sports. This has great practical value in coaching and player development, and can feed questions back into basic research as well.

When I look at well-conceived and executed research, I look in particular for if and how the “representative tasks” devised by researchers might be re-purposed for testing and training. This study certainly provides methods to test and train goalkeepers in VR.*

RESPONSE

We wholeheartedly agree. In fact, while we were completing this study one of us (CC) started a company to pursue this application of virtual reality. To underscore this potential of VR simulators, we now mention this in the Discussion (lines 567-570).

*2.0: Reviewer #2: This paper investigated an interesting topic; visual control of action in football goalkeeping during a free kick with and without a wall. Whilst the paper is generally well written, several sections can be improved as follows.*

RESPONSE

We thank the reviewer for their comments. We will summarize our responses to the six main points below.

*2.1: First, a clearer theoretical framework is needed of how early and late visual information maps to guide actions with links to relevant analyses.*

RESPONSE

Our original manuscript indeed did not discuss a theoretical framework for early and late visual information with respect to the control of action. The reason for this is that our study did not concern what explicit information is blocked by the wall; our study addressed the higher-level question of whether lack of early information affected performance. However, in response to this comment we do now allude to work that relates to the distinction between (the use of) early and late information in the Discussion (lines 494-512).

*2.2: Second, the manuscript is lengthy, difficult to follow in the disjointed presentation of experiments 2 and 3. In addition, I was not convinced by the use of novice participants in experiments 2 and 3. I would limit the paper to experiment 1.*

RESPONSE

We have rewritten large parts of the manuscript with the aim to improve coherence of the presentation. Among others, this comment motivated us to only present the first experiment in the main paper. All Methods and Results for Experiments 2 and 3 have been moved to the Supporting Information. Only in the Discussion do we shortly allude to the consistency of the effects across variants of the experiment, while noting that the absence of skilled goalkeepers in those experiments limits the practical implications that can be drawn for those scenarios.

*2.3: Third, participants need to be accurately categorised as skilled, not experts.*

RESPONSE

In response to this comment as well as comments by the other reviewers, we have changed the names of our groups to “naïve participants” (i.e., they are not goalkeepers) and “skilled goalkeepers”. Moreover, in response to Reviewer 3’s comment concerning the exact classification, we provide a breakdown of the level of expertise of our skilled goalkeepers (based on Baker et al., 2015; in terms of the highest level of competition played in) in the Methods/Participants section.

*2.4: Fourth, use of VR technology as reported in the methods is impressive, but I am confused by the omission of kicker kinematics.*

RESPONSE

We replied to this in detail below, in response to comment 2.8.

*2.5: Fifth, the authors should conduct further work using truly expert goalkeepers before they recommend omission of the wall during free kicks. The wall can influence the kicker’s strategy and difficulty of shot execution.*

RESPONSE

We tried to be careful in our wording, because we are aware that our results do not provide a basis for a general advice to remove the wall. For this reason, we indicated goalkeepers should *consider* removing the wall and only in specific situations (where the wall could be argued not to affect the kick). However, we now appreciate that our wording was not careful enough. Practical impact (the question whether the wall affects performance in skilled players) was one aim of our study; however, we would argue that practical impact is not limited to truly expert players (although it would evidently be most visible in professional football). This concern motivated us to expand our treatment in the Discussion of what our results do and do not mean for skilled football players in practice (lines 523-557).

*2.6: Finally, please add a limitations section based upon my comments. More specific details are below.*

RESPONSE

We have added a section in the Discussion on these issues, as alluded to in our responses to comment 2.5. However, we did not call this a ‘Limitations Section’, since these factors do not affect the primary aim of the study.

*2.7: Specific Comments Line 29; I was not convinced by the use of novices in experiments 2 and 3. For instance, the authors hypothesise an expertise effect (line 78-79). Goalkeeping is a highly specialised skill, so in order to make practical recommendations the actual expert or skilled performer needs to be studied. Introduction starts with a professional football example, but does not explicitly state how true expert (or skilled) goalkeepers use visual information to perform to a superior level compared to less-skilled goalkeepers. This is relevant to the hypothesis (line 78-79). Please make this clear in the introduction.*

RESPONSE

Besides pragmatism (the wider availability of naïve participants), the motivation for including only naïve participants in the additional 2 experiments motivated by the large alignment between the patterns shown by goalkeepers and naïve participants reported by Dessing & Craig (2010) and apparent in nearly all the patterns reported for our first experiment. It is therefore not unlikely that the findings of the additional experiments would generalize to actual goalkeepers. Nevertheless, this comment as well as other comments (e.g., concerning the somewhat disjointed nature of the presentation) motivated us to focus the manuscript on the first experiment and to move any treatment of methods and results of the additional experiments to the Supporting Information. Importantly, we do still allude to the experiments and the general consistency of the findings for naïve participants in the Discussion (see line A), while stating that verification of the explicit effects for skilled goalkeepers awaits empirical confirmation (see lines 517-520). In response to Reviewer 1, we explained in detail why/that although our aim was to focus on the effects of the wall, we now do present the one statistical test of the effect of expertise, in line with the statement in the Introduction (now lines 73-76). We now refer to studies suggesting expertise comes with superior visual, motor, and visuomotor skills, to support this hypothesis (lines 75-76).

*2.8: Line 55; sure, but if the wall is omitted the goalkeeper would normally see the kicker’s kinematics. This needs to be listed as a limitation. Also, goalkeepers position themselves to a side of the wall to pick-up kicker kinematic cues.*

RESPONSE

The reviewer is right that in practice the wall also occludes the kicker’s kinematics (as was/is also stated in the abstract/Introduction [lines 17, 80-85]). Since previous studies focused on the effects of visual occlusion of the ball trajectory on catching movements (see Introduction), we aimed to assess whether these lab-based observations generalized to a practical scenario. In this particular study, we thus only focused on the effect of visual occlusion by the wall of the initial ball trajectory. We now more explicitly discuss this in the Introduction (Lines 65-66, 80).

It is currently not known to what extent goalkeeping for free kicks relies on information about kicker kinematics. Certainly, due to longer flight times it may be expected to be less critical to performance than for goalkeeping in penalty shot scenarios (where time constraints dictate goalkeepers must mainly rely on kicker kinematics, at least for the sideward component of the movement [Higueras-Herbada et al., 2020]). We appreciate that this was not optimally captured by our formulation “goalkeeper performance in this context does not *critically* depend on seeing the kicker”. We have thus reworded this to “Free kicks allow more time for online control based on ball flight information, which means balls can be stopped even when the kicker is not seen at all, as evident from our previous studies”. We expect that during free kicks, aspects of kicker kinematics certainly inform the goalkeeper on the expected direction of curve (e.g., a left-footed player (mostly) will generate rightward curve [and vice versa]) and possibly the required direction of movement (as the reviewer suggests). However, such effects likely would mean performance without a wall would be even better; had we included kicker kinematics, the benefits of removing the wall might have thus been more dramatic.

When including a kicker, we would not have had as much control over the details of the ball trajectories, since we would deem it essential to have alignment between the kicker kinematics and ball trajectories (for reasons of practical representativeness). This is the choice one has when using virtual reality sports simulators: either use simulations, which – at least at present - cannot include fully realistic kicker kinematics, or recordings (of kicker and ball). Here, we used the former, but that does not mean we are not also interested in the benefits of seeing the kicker kinematics for the goalkeeper. In fact, before COVID-19 hit, we were about to start recordings of kicker kinematics and associated ball trajectories for a future VR study.

Besides changing the formulation referred to above, we followed the reviewer’s suggestion by including a more extensive treatment of the potential effects of design choices that meant our simulator did not perfectly match a real-life free kick scenario (including the absence of a kicker avatar and the fact that we prevented freedom for participants to choose their initial position) (lines 524-557).

*2:9 Also, goalkeepers position themselves to a side of the wall to pick-up kicker kinematic cues.*

RESPONSE

As the reviewer notes, in real free kick scenarios, goalkeepers often position themselves (or the wall) to see the kicker; this was and is mentioned in the Discussion (lines 537-557). The central question concerned the general effects of not seeing the initial ball trajectory; freedom to move could influence the amount/duration of occlusion. Moreover, if goalkeepers position themselves more to one side of the goal, they leave open the other side of the goal, which likely influences performance. Because both effects likely increase variability in our data, we opted not to allow for such freedom to standardize conditions across participants (for the sake of experimental control / statistical power). We have expanded our discussion of this aspect of our design in the Discussion (lines 537-557).

*2.10: Line 65-72; this section is where a model of early and late visual information that maps onto actions is of value to the manuscript (see Morris-Binelli & Müller, 2017).*

RESPONSE

This section of the Introduction has been slightly changed; as a result, we felt this is not the appropriate place to discuss this work; we do discuss it in the Discussion though (lines 494-512).

*2.11: Line 78-79; please clearly discuss literature on true expert (or skilled) goalkeepers in order to support this hypothesis.*

RESPONSE

Related work is now discussed (lines 73-76).

*2.12: Line 80-81; I was not convinced as to why the analyses were separated by goalkeeper expertise, when the hypothesis clearly states as expertise effect. A factorial ANOVA is needed.*

RESPONSE

This comment aligns with comment 1.4 of Reviewer 1, in response to which we have motivated why we tested the effects separately for naïve participants and skilled goalkeepers. We also describe that we now provide the test associated with the proposed expertise effect (lines 73-78, , ,). A factorial ANOVA is not needed. The proposal mentioned on lines 78-79 (now 73-78) requires a comparison of the effect of the wall on performance between naïve participants and skilled goalkeepers (which is the test we report). A factorial ANOVA would test for many more effects, but we did not propose any other effect of expertise and thus do not test for it (motivated by the desire to minimize the number of statistical tests conducted). Rather than replacing any of the tests we conducted, we present this as an additional test in the revised manuscript. We think the reviewer refers to the approach of testing for the main/overall effect of the Wall (across Expertise levels) and how this effect is modulated by Expertise (/differs between Expertise levels – statistically related, but not equivalent to the Expertise x Wall interaction in a factorial test [in fact, our single, direct test of this modulation has a higher statistical power]). However, this approach would not afford conclusions directly relevant to practical impact. Tests of the effect of the Wall across Expertise levels and of how the effect of the Wall is modulated by Expertise (/differs between Expertise levels) would not establish statistically whether either of the groups themselves were significantly affected by the wall. This knowledge is essential for any evidence‑based suggestion to sports practice (which is mainly for the skilled group). Another reason why a factorial test is not best is the fact that our key descriptor of performance (AE) cannot be assumed to be normally distributed. A non-parametric test is needed and there is no established non‑parametric variant of a multi-way mixed-design ANOVA, which (among others) means there is no way to test the main effect of the wall (across Expertise levels).

Based on these considerations (see also our reply to Reviewer 1’s comment 1.4), as mentioned we decided to test for the effect of Expertise on AE (i.e., how the effect of the wall differs between Expertise groups). No other tests of Expertise were presented (since these were not predicted/were not the focus of this study). In the revised manuscript, we have updated our description of the proposed Expertise effect (lines 73-78), we describe the statistical test in the Methods (lines 312-313), its outcome in the Results (lines 354-356), and in the Discussion (lines 468-470, 475-476).

*2.13: Line 97; given the available technology and space, I am not sure why the kicker’s kinematics was not also simulated. This needs to be listed as a limitation. Both kinematic and ball flight information would make the task more presentative (see Pinder, Davids, Renshaw, & Araújo, 2011).*

RESPONSE

This comment aligns with comment 2.8. While we indeed have the space and technology to record kicker kinematics, it just was not the focus of this study. As indicated in response to comment 2.8, our focus was on occlusion of the initial ball trajectory. The task would only be representative if the kinematic and ball flight information were aligned (i.e., based on recordings of both the kicker kinematics and actual ball trajectory). This leaves less experimental control of the parameters of the ball trajectory. It is clearly a trade-off between scientific control and practical representativeness. We would argue that this is not a scientific limitation, but a potential limitation for practical relevance (although as we argue in our response to comment 2.8. it is not unlikely that the relative effects of the wall would be bigger [i.e., performance without a wall better] when kickers are included. This possibility is interesting, and we will be able to address this in our upcoming study (referred to in comment 2.8). We stress that this in no way invalidates our current study, which focused on the effect of visual occlusion of the initial ball trajectory. In response to this comment, we have updated our discussion of the differences between our simulator/task and goalkeeping in real free kick situations in the Discussion (lines 523-557). To emphasize the focus on visual occlusion of the initial ball trajectory, we now also refer to it in our updated title of the manuscript:

*“A goalkeeper’s performance in stopping free kicks reduces when the defensive wall blocks their initial view of the ball”*

*2.14: Line 219; why is skill level not part of these predictions?*

RESPONSE

See our responses to comment 1.4 of Reviewer 1 and comment 2.12 of this reviewer.

*2.15: Line 244; not sure why success rate could not be analysed using a statistical test?*

RESPONSE

This comment relates to comment 1.5 of Reviewer 1. As indicated in our response to that comment (and in other responses), we had the desire to limit the number of statistical tests to those most relevant, to avoid inflation of the chance of making Type 1 errors (while keeping statistical power as high as possible). The measures of AE and Success Rate are largely redundant (i.e., highly related); we opted to perform the statistical tests we judged to be most powerful/sensitive, which meant testing AE and not Success Rate.

However, because Success Rate is a practically relevant variable (i.e., it is observable on the field without tools), we decided to highlight visually (in the insets in the pertinent panels) how the different comparisons for AE break down for Success Rate (without presenting statistical comparisons). We treat this as a descriptive statistic. In the revised manuscript we have clarified this motivation and qualification of the Success Rate variable (see 235-242).

*2.16: Line 264-267, 291-296; the model mentioned above would help in the interpretation of this data in discussion section (theoretical implication).*

RESPONSE

In response to this comment, we have adjusted parts of our Discussion in line with the suggestion of the reviewer (lines 494-512).

*2.17: Line 310-311; I do not understand why a main analysis would not include skill level as a factor? If not relevant, only study the skilled group, which would provide a stronger argument for your practical recommendation in due course.*

RESPONSE

We believe our responses to comments 2.7 and 2.12 have answered this. We now do test how the effect of the wall on performance differs between expertise groups.

*2.18: Line 324-326; why present the figure if you state its content cannot be appreciated?*

RESPONSE

We believe this comment does not align with our motivation for including this figure. We agree that if our intention was to illustrate the kinematics for conditions with and without a wall, this figure would not add much. Our intention, however, was to illustrate raw data, to give readers a feel for what the trajectories look like. The remark concerning the effect of the wall not being appreciable from the figure was meant to emphasize that the wall does not qualitatively affect the trajectories. In the revised manuscript, we have made this motivation clearer (lines 322-327).

*2.19: Line 339; effect of the wall relative to skill level performance seems the missing analysis.*

RESPONSE

The results of this analysis has now been included (lines 354-356; see also our responses to comments 1.4, 2.7, 2.12).

*2.20: Line 377; this section onwards is relevant to the model of visual control of action mentioned earlier.*

RESPONSE

In response to this comment, we have adjusted parts of our Discussion in line with the suggestion of the reviewer (lines 494-512).

*2.21: Line 407 para; reference to Table 1?*

RESPONSE

Reference to Table 1 is now included (line 353), also in the other sections.

*2.22: Line 488; my understanding is that goalkeepers do not stand directly behind the wall. This needs to be discussed as a limitation.*

RESPONSE

This comment relates to comment A. We now discuss this aspect of our design in relation to real-life free kick scenarios (lines 537-557).

*2.23: Line 489; theoretical implications.*

RESPONSE

Change made within the Discussion. (lines 494-512)

*2.24: Line 499-500; is wireless VR an option?*

RESPONSE

Yes. Wireless VR only became available while we were conducting the experiments. We now do have the wireless VR option and will use this in future experiments when needed. It is likely that wireless VR will afford safe experiments with more eccentric passing distances and diving; we are currently planning experiments that do include this option. We discuss this in the revised manuscript (lines 550-552).

*2.25: Line 511; expertise effects are discussed, but the introduction content, hypotheses, and analyses related to expertise need to be more clearly aligned and presented.*

RESPONSE

In fact, no statistical expertise effects are discussed here. This sentence matter-of-factly states that naïve participants showed an effect and that skilled goalkeepers did not; we do not refer to a statistical difference between the groups. This formulation is in line with the separate testing of the effects for both groups (see responses to comments A, B, C for our extensive motivation for not testing all novice‑expert differences). However, we now appreciate that ‘Interestingly’ is poorly chosen in this sentence and could give the impression that an Expertise effect is referred to. In response to this comment, we have thus replaced this sentence with (line 486-490):

“In naïve participants, these early biases ultimately influenced interception performance for these trajectories; our skilled goalkeepers showed the same early biases, but no significant biases were found in their interception performance for congruent trajectories. Thus, the early movement is affected more consistently by the wall for congruent trials and, tentatively, only skilled goalkeepers were able to correct for this during their movement.”

*2.26: Line 537 para; this section does not discuss the mechanism of visual control of action. See earlier comments.*

RESPONSE

As indicated in response to earlier comments, we have included this work in our Discussion (lines 494-512).

*2.27: Line 552 para; as mentioned earlier, further work is needed with truly expert goalkeepers before strong recommendations can be made. If the wall is omitted, kicker kinematic cues would also become important. This should be mentioned.*

RESPONSE

We agree that inclusion of truly expert goalkeepers would broaden the range of players for which recommendations can be made. We disagree, however, that only recommendations for the top 1% of players (a hypothetical number) would be worth making – this conflates media attention with practical impact. As the reviewer indicates, omission of the wall means that (more) kinematic cues likely become available to the goalkeeper; in our response to comments 1.7 and 2.8 we argue that this particular benefit likely amplifies the effect of the wall on performance (i.e., increasing the Wall-NoWall difference by improving performance without the wall [at least for the same free kick trajectories]). We now discuss these issues in more detail in the Discussion (lines 525-536).

*2.28: Line 578; the wall can influence the strategy of, and challenge, the kicker.*

RESPONSE

We agree with the reviewer that kickers are likely influenced by the wall. One influence could be the aiming strategy: it has been suggested that skilled free kick takers use the wall (i.e., its visual configuration relative to the goal) to aim their shot (anecdotal evidence was included in a link in the manuscript; see [https://www.skysports.com/football/news/11095/11310892/watch-gianfranco-zola-joins-jimmy-bullard-for-a-free-kick-masterclass](https://www.skysports.com/football/news/11095/11310892/watch-gianfranco-zola-joins-)). This makes sense scientifically as well: it is known that allocentric (i.e., relative position) information is used for interactions with objects in 3D space (e.g., https://www.ncbi.nlm.nih.gov/pmc/articles/PMC5390010/). We appreciate that the formulation used in this sentence does not reflect our intention, namely that it refers to expert free kick takers who can avoid the wall with shots that are hard (i.e., short flight time) and (evidently) on goal. We have rephrased this part of the Conclusion to clarify this (line 571-579).

Reviewer #3: This manuscript sets out to assess the effects of visual occlusion by the wall on goalkeeping performance from free kicks which is practically relevant as the free-kicks provide a goal scoring opportunity. The use of an immersive, interactive virtual reality system in the current study allows for a significant degree of experimental control. Please see attachment for reviewer comments.

**Comment to Authors**

**General Comments**

This manuscript sets out to assess the effects of visual occlusion by the wall on goalkeeping performance from free kicks which is practically relevant as the free-kicks provide a goal scoring opportunity. The use of an immersive, interactive virtual reality system in the current study allows for a significant degree of experimental control.

3.1: While the authors have implemented a high degree of scientific rigour there are a number of concerns that need to be addressed. The most notable concerns relate to the generalisability of this research to real-world context. Despite the high degree of experimental control, this research fails to significantly take into consideration the potential actions of the opposing team members which would significantly impact the inclusion/removal of a defensive wall.

RESPONSE

The reviewer is correct in his assessment that our study does not address/take into account several strategies that may be allowed by removing the wall in actual free kick scenarios. However, this comment slightly misrepresents the aim of our study. Our scientific aim was to assess whether the frequently reported effects of (early) occlusion on interception performance generalize to the practical scenario of interception of free kicks by goalkeepers. This may have practical implications, but we anticipated that the experimental control sought in this study meant that, at this stage, no solid *general* practical advice (i.e., to always remove the wall) could follow from our study (unless extreme results were found). For this reason, pursuing this practical generalizability was never the *primary* aim; we certainly consider it a potential outcome of the overarching (PhD) project. We appreciate that this was not made clear enough in the original manuscript and believe our revised presentation in the Introduction (lines 60-63, 65-66) and Discussion (lines 463-464) more clearly sets out these aims and describes what can and cannot be concluded from this study.

3.2: The removal of the free kick taker from the virtual reality system is also a point of contention, as the position of the free kick taker prior to taking the free kick can provide the goalkeeper with contextual information on potential arrival destinations. In addition to this the consideration for the action capabilities of the free kick taker if the wall was indeed removed do not seem to be fully addressed. If, as suggested in the conclusion of this paper, the goalkeeper removed the wall the free kick taker may choose a significantly different free kick style presenting trajectories different to those utilised in this study.

RESPONSE

We agree that it is an interesting question to what degree and how goalkeepers in free kick scenarios use information about the kicker kinematics to guide their actions. Moreover, we agree that without a wall a different type of shot is possible, which might be more challenging for the goalkeeper to stop. Not showing a free kick taker is one of those aspects we chose for scientific control (see previous comment/response). As explained in response to comment 2.8, however, it is quite likely that the view of the kicker movements when omitting the wall will in fact exaggerate the effect of the wall (by improving performance in conditions without a wall), at least for the same free kick trajectories. If removal of the wall motivates a more challenging shot, a benefit of seeing the kicker would still be there – it is unknown how any benefit compares to potential negative effects of the shot being more challenging. The interplay of a multitude of factors partly underscores the point made by the reviewer: no general advice is possible based only on the present findings. Indeed, we did not provide such general advice. We only identified a very specific situation for which removal of the wall could at this stage be sensibly suggested based on our findings. In our Conclusions, we stated that goalkeepers could *consider* removing the wall when faced with a free kick taker who hardly ever is negatively affected by the wall. The ‘consider’ was meant to involve a judgment concerning potential negative effects of removing the wall, such as the mentioned different free kick style. We did not make this explicit, which we should have. Based on the reviews, we have now further specified/constrained this situation and consistently state, in the Introduction and Discussion (including the Conclusions) that our findings cannot be taken to suggest a blanket removal of the wall. We have expanded our treatment of what our findings can and cannot say about real goalkeeping (lines 522-593).

3.4: This manuscript also needs to be restructured as content that should be in the introduction, methods, and discussion sections often features in the results section. It may be worth considering restructuring the manuscript to sequentially present the three experiments. For an example of this structure style please see Müller, Abernethy & Farrow - DOI: 10.1080/02643290600576595

RESPONSE

Based on comments made by the other reviewers, we have in fact decided to move all details concerning the additional two experiments to the Supporting Information. The key motivation for this was the fact that no skilled goalkeepers were included in these additional experiments, which means conclusions concerning practical implications would be based on inference/estimation, rather than actual evidence. There thus are no issues anymore with respect to sequential presentation of experiments.

**Specific Comments**

***Introduction***

3.5: L49 – While I don’t disagree with the quality of the free kick example given, taken by Kieran Trippier, I find the reference to players as *“Professional free kick takers”* unusual. Kieran Trippier, the primary example in this research, is a professional football player but the classification as professional free kick taker needs to be reconsidered as he has only scored 5/6 free kicks over the course of 10 seasons as a professional footballer.

RESPONSE

We agree with this comment and have reworded this to say:

“Elite players can shoot free kicks with speeds in excess of 28m/s” (lines 48-49).

3.6: L52-55 – To state that goalkeeper performance does not *critically* depend on seeing the free kick taker is a misleading statement. Yes, as identified by the authors online control which could be referred to as prospective control) can be an effective method for goalkeepers to utilise but that does not mean you can disregard the important role of predictive information (see Panchuk and Vickers 2009 [10.1080/02640410903156449](https://doi.org/10.1080/02640410903156449)).

RESPONSE

Our use of ‘critically’ was meant to indicate that interception does not become impossible without seeing the kicker. We in fact were not referring to an interception strategy; our study cannot distinguish between predictive and online/prospective control. We did not mean to imply goalkeepers do not use information about kicker kinematics; we would fully agree with the reviewer that if beneficial, such predictive information would be used by goalkeepers. In response to this comment, we have reworded the formulation referred to (lines 52-57) and expanded our discussion on the potential use of kinematic information in the Discussion (lines 523-536).

3.7: L60-61 – This studies cited to identify that catching performance is negatively impacted when less time is available are conducted using one-handed catching tasks, which differentiate significantly from saving a free-kick, the performance measure used in this study. This needs to be clearly stated.

RESPONSE

This is an important point, which indicates that we did not enough describe our aims clearly. Our primary aim refers exactly to this difference: to examine whether the previously observed patterns/effects for laboratory-based one-handed catching movements generalizes to the more practical interception scenario of (one- and/or two-handed) goalkeeping (for which such effects might have practical implications). We have clarified this formulation to more clearly specify our aims (lines 60-63).

3.8: L69-71 | L78-79 It would be beneficial to directly address what is hypothesised in the introduction more clearly in the discussion section.

RESPONSE

In line with the reviewer’s suggestion, we now more clearly list our hypotheses at the end of the Introduction (lines 67-78) and discuss these more coherently in the Discussion (lines 463-476).

***Methods***

3.9: L91 – Please clearly identify that it is age you are referring to here.

RESPONSE

Changed as requested (lines 93-94).

3.10: L92-93 – This is a low threshold of experience to use the term expert. I would suggest using the taxonomy for skill in sport outline in the Routledge Handbook of Sport Expertise by Baker and Farrow in chapter 13 P147 as a guide – see below.

RESPONSE

We agree with the reviewer; no formal classification was used. Our expert goalkeepers in the linked taxonomy would range from Basic to Advanced. Evidently, we would have wanted to recruit a wider range of skilled goalkeepers (especially even more skilled ones), but this proved challenging in our environment. In response to this comment, we have renamed our expert goalkeepers to ‘Skilled goalkeepers’ and we specify their classification within Baker et al.’s taxonomy (lines 98-99). Similarly, we renamed our ‘novice goalkeepers’ to ‘naïve participants’ (nb., the latter participants really were not goalkeepers).

3.11: L142-146 – The introduction of dummy trajectories is an excellent addition to the study design and provides valuable variability.

RESPONSE

We thank the reviewer for emphasizing this aspect of our design. Please note that in response to comment 1.4, we have expanded our explanation for including the dummy trajectories (lines 149-155).

3.12: L169-171 – Is there a reason for this hand position. If so, it would be beneficial for readers if this was explained.

RESPONSE

There indeed was a reason for this hand position. We wanted to standardize the initial hand position without using physical constraints. Another benefit of this choice was that it meant the virtual hands were in view before each trial, which we felt might benefit the immersive experience (although we have no evidence for this). In response to this comment, we have expanded motivation for using this initial position (lines 180-184).

3.13: L181-182 – On L143 you state that there are 90 unique dummy ball trajectories, 4 trials of each would equate to 360 trials not 180. Can you clarify the number of trials please?

RESPONSE

We thank the reviewer for noting this inconsistency. There were in fact 45 unique Dummy trajectories (3 final heights, 5 final sideward positions, 3 curve conditions) which were used both with and without a wall. Those 90 conditions were repeated twice, for a total of 180 trials. We have changed our description on lines 151 and 192-193 to indicate there were 45 unique dummy trajectories.

***Research Question***

3.14: Is this a sub-heading under data analysis? Is there a reason research questions is placed in this part of the manuscript and not at as null hypothesis at the end of the introduction section?

RESPONSE

We agree that any extensive treatment of the research questions is better presented in the Introduction. We have thus expanded the treatment of research questions in the Introduction (lines 64-79) and removed this list/heading from the Methods.

3.15: L222 – Movement initiation can be a deceiving measurement when we consider the potential for individual differences in control strategies (for example see Ijiri et al 2014 – doi: 10.1080/02640414.2014.946073). Movement-onset (initiation) and movement duration can be very different among participants. This, at the very least, needs to be acknowledged as a limitation in the manuscript.

RESPONSE

While we agree that such individual differences may be relevant for some situations, our predictions concerned the effect of the wall on movement initiation. The prediction is very general: visual information becomes available later, which should delay movement initiation. Although the size of the delay could of course vary depending on the strategy, the prediction of a delay per se is independent of the control strategy used. Our study was not designed to assess differences in strategies between participants and the predictions concerning the delayed movement initiation were made across flight times (~forward ball speeds). We would not classify this as a limitation of our study. Moreover, our data also do not support any major differences in the delay across participants. We have thus decided not to address this concern.

***Statistics***

3.16: L304 – I believe this sentence is missing the word “be” - AE to be non-normally.

RESPONSE

The reviewer is correct; change made (line 309).

3.17: L311 – Was there a reason for not directly comparing groups?

RESPONSE

All reviewers commented on this. Our most extensive response to this can be found in our responses to comments 1.4, 2.7, 2.12.

***Results***

*3.18: L317 – The* *results section contains a lot of repetition and descriptive discussion content. The results should only report the finding of the analysis with little to no interpretation. Amend throughout the results section and simply report the findings.*

*L318-321 | L374-378 | L407-409 | L412-414 | L429-437 – These are prime examples of what I am referring to. The results section is not a place to restate the aims of the research study, provide comparisons to other research studies or provide a rationale for why particular measurements were conducted.*

RESPONSE

We have, where possible, removed this content from the Results section. However, we have retained some (shortened) description in the Results section for the sake of readability and clarity. Some analyses were motivated from the data (the follow-up analyses) and we believe it greatly helps the reader to tell the story of the data by discussing which aspects motivated which analyses (rather than continuously referring back the Methods section). Thus, we have compacted our Results section by moving most of the motivation to the Methods section and only shortly summarizing it in the Results section.

***Discussion***

*3.19: L500-502 – Restructure this sentence for clarity.*

RESPONSE

Done. Sentence now reads (lines 555-557):

“Indeed, we expect performance to be affected even more by the wall for such more challenging free kicks, since a delayed initiation would be more problematic if a larger distance must be covered (see also [13])”

*3.20: L518-522 – Main Experiment and first experiment are used interchangeably here. Please choose a term and amend throughout for consistency.*

RESPONSE

Thank you for highlighting this. Our decision to move all information concerning the additional 2 experiments to the Supporting Information means this should not be relevant anymore. Any reference to ‘Main Experiment’ as well as ‘First experiment’ has been removed. Only the Supporting Information makes reference to “Main Experiment” in comparing aspects of the 3 experiments.

*3.21: L544 – Is there a need for “for experts they did not” to be in brackets here?*

RESPONSE

Removed.

*3.22: L555 – “Flipside” is two words. It may be worth changing this to term to disadvantage/drawback.*

RESPONSE

We agree; “drawback“ is now used (line 541).

*3.23: L556-561 – Despite identifying that it is something that needs to be examined in future studies, to suggest that free kick takers or soccer/football players rely on the wall to take a free kick and that removing the wall would make the free kick more difficult seems counter-intuitive. The removal of the wall may allow free kick takers to act upon a number of action capabilities available to them.*

RESPONSE

The suggestion that expert free kick takers use the wall (i.e., its visual configuration relative to the goal) is based on anecdotes (<https://www.skysports.com/football/news/11095/11310892/watch-gianfranco-zola-joins-jimmy-bullard-for-a-free-kick-masterclass>) and makes sense scientifically as well (i.e., aiming at objects in 3D space based on allocentric (i.e., relative position) information). Based on the reviews, we have updated this section, which should also clarify what we meant to highlight in the lines the reviewer refers to (lines 571-579).

References

Higueras-Herbada, A, Lopes, J. E., Travieso, D., Ibáñez-Gijón, J., Araújo, D., Jacobs, D. M. (2020). Height After Side: Goalkeepers Detect the Vertical Direction of Association-Football Penalty Kicks From the Ball Trajectory. *Front Psychol, 11*:311.
